# TLR3 Serves as a Prognostic Biomarker and Associates with Immune Infiltration in the Renal Clear Cell Carcinoma Microenvironment

**DOI:** 10.1155/2021/3336770

**Published:** 2021-09-06

**Authors:** Guodong Liao, Jia Lv, Alin Ji, Shuai Meng, Chao Chen

**Affiliations:** ^1^Department of Urology, The First Affiliated Hospital, College of Medicine, Zhejiang University, Hangzhou City, Zhejiang Province 310006, China; ^2^Department of Urology, Zhejiang Provincial Peoples' Hospital, Peoples' Hospital of Hangzhou Medical College, Hangzhou 310014, China; ^3^Department of Urology, Affiliated Hangzhou First People's Hospital, Zhejiang University School of Medicine, Hangzhou City, Zhejiang Province 310006, China

## Abstract

**Background:**

Clear cell renal cancer (KIRC) is one of the most common cancers globally, with a poor prognosis. TLRs play a vital role in anticancer immunity and the regulation of the biological progress of tumour cells. However, the precise role of TLRs in KIRC is still ambiguous.

**Methods:**

Various bioinformatics analysis and clinical validation of tissues were performed to evaluate the prognostic value of TLRs and their correlation with immune infiltration in KIRC.

**Results:**

The expression of TLR2/3/7/8 was increased at both mRNA and protein levels in KIRC. TLRs in KIRC were involved in the activation of apoptosis, EMT, RAS/MAPK, and RTK pathways, as well as the inhibition of the cell cycle and the hormone AR pathway. Drug sensitivity analysis revealed that high expression of TLR3 and low expression of TLR7/9/10 were resistant to most of the small molecules or drugs from CTRP. Enrichment analyses showed that TLRs were mainly involved in innate immune response, toll-like receptor signalling pathway, NF-kappa B signalling pathway, and TNF signalling pathway. Furthermore, a high-level TLR3 expression was associated with a favourable prognosis in KIRC. Validation research further confirmed that TLR3 expression was increased in KIRC tissues, and high TLR3 levels were associated with poor overall survival. Moreover, TLR3 in KIRC showed a positive association with an abundance of immune cells, including B-cells, CD4+ T-cells, CD8+ T-cells, macrophage, neutrophils, and dendritic cells, and the expression of the immune biomarker sets. Several TLR3-associated kinase, miRNA, or transcription factor targets were also identified in KIRC.

**Conclusion:**

Our results indicate that TLR3 serves as a prognostic biomarker and associated with immune infiltration in KIRC. This work lays a foundation for further studies on the role of TLR3 in the carcinogenesis and progression of KIRC.

## 1. Introduction

Renal cell carcinoma (RCC) is one of the most common malignancies globally, accounting for 2.4% of all cancer diagnoses [1]. In China alone, each year, an estimated 66,800 people are initially diagnosed with RCC, and 234,000 patients lose their life due to this disease [2]. Clear cell renal cell carcinoma (KIRC or ccRCC) is the most common and aggressive subtype of RCC, accounting for more than 80% of all cases [3]. In recent years, significant progress has been made in the treatment of renal cell carcinoma with the advancements in targeted therapy and immunotherapy, improving the therapeutic effect and the quality of life of patients to a certain extent [1]. However, many KIRC patients still develop drug resistance and progressive diseases, increasing the risk of a poor prognosis [4]. The 5-year overall survival (OS) of stage-IV KIRC patients is less than 10% [5, 6]. A recent study revealed that the immune cell response is significantly associated with the prognosis in KIRC [7]. Moreover, immunotherapy has been suggested as a promising treatment for metastatic KIRC [8]. Therefore, clarifying the correlation between KIRC and immune infiltration and identifying immune-associated markers for the prognosis for KIRC are particularly necessary.

Toll-like receptors (TLRs) are a family of transmembrane receptors that recognise various pathogens and play a vital function in inflammation related to molecular patterns by mediating NF-*κ*B signalling [9]. A total of ten members of TLRs (TLR1-10) have been found in the mammalian genome [10]. TLRs in the tumour microenvironment (TME) are expressed not only by innate and adaptive immune cells but also by stromal cells, such as fibroblasts, endothelial cells (EC), and tumour cells [11]. Accumulating studies suggest the importance of TLRs in anticancer immunity and the regulation of the biological progress of tumour cells [12, 13]. Moreover, TLRs have been suggested as prognostic biomarkers in many cancers and other diseases, including TLR1/2/4/8 for colorectal cancer [14], TLR4/7 for breast cancer [15], and TLR5 for gastric cancer [16]. However, whether TLRs could be prognostic biomarkers and their correlation with immune infiltration in the KIRC microenvironment have not been clarified yet.

Thus, the current study was conducted to detect TLRs expression and explore its prognostic value in KIRC. The correlation between ATLRs and immune infiltration was also analysed. Our results may provide additional evidence for the role of TLRs in KIRC and their association with immune infiltration.

## 2. Materials and Methods

### 2.1. Expression Level Analysis in Oncomine™, GEPIA, and UALCAN

The expression of TLRs in KIRC was detected in the Oncomine (https://www.oncomine.org) [17], GEPAI (http://gepia.cancer-pku.cn/) [18], and UALCAN (http://ualcan.path.uab.edu) databases [19]. In the Oncomine database, we detected the mRNA expression of TLRs in different tumours, including KIRC with a *p* value of 1E−4, a fold change (FC) of 2, and gene ranking of top 10% as the threshold [20]. To further verify the mRNA expression of TLRs in KIRC, we detected their level using the Cancer Genome Atlas (TCGA) data set in GEPIA with a *p* value of 0.05 as the threshold. Moreover, we then used UALCAN to explore the protein expression in KIRC with data from the Clinical Proteomic Tumor Analysis Consortium (CPTAC). We also performed different subgroup analyses based on gender, age, tumour grade, and cancer stages at both mRNA and protein levels to investigate the correlation between TLR3 expression, and the clinicopathologic features of KIRC patients. A value of *p* < 0.05 indicated that the difference was statistically significant.

### 2.2. Survival Analysis Using Kaplan–Meier Plotter and OSkirc

We used Kaplan–Meier plotter (https://kmplot.com/) [21] and OSkirc (http://bioinfo.henu.edu.cn/KIRC/KIRCList.jsp) to explore whether TLRs could act as prognostic biomarkers of KIRC [22]. High/low ACE2 expression patients were identified by the median value of TLRs expression and a *p* value of 0.05 as the threshold. In the Kaplan–Meier plotter, the overall survival (OS) curve of TLRs in KIRC was drawn by Kaplan–Meier estimator using the TCGA KIRC data set (*N* = 537). Subgroup prognosis analysis based on different clinicopathologic features and immune cells was also performed to examine how TLRs affect the prognosis of KIRC patients. In OSkirc, the OS of TLRs in KIRC were analysed with the GSE29609 data set. A value of *p* < 0.05 indicated that the difference was statistically significant.

### 2.3. Validation of the Expression and Prognosis Value of TLR3 in KIRC

A total of 30 KIRC tissues and normal kidney tissues were obtained from patients who underwent tumour resection. All patients provided informed consent. Histological diagnosis and tumour grade were assessed by three experienced pathologists following the guidelines set by the 2010 American Joint Committee on Cancer Staging system. Patients did not receive any treatment before operation. Total RNA of endometrial cancer tissues and normal endometrial tissues was extracted with TRIzol reagent. The synthesis of cDNAs corresponding to the mRNAs of interest depended on PrimeScript RT-polymerase (Vazyme). All reactions were performed using SYBR-Green Premix (Vazyme) with specific PCR primers (Sangon). Glyceraldehyde-3-phosphate dehydrogenase was used as an internal control. The 2−ΔΔCt method was employed to calculate fold changes. Primer sequences were as follows: GAPDH, forward: GCACCGTCAAGGCTGAGAAC; reverse: TGGTGAAGACGCCAGTGGA and TLR3, forward: CCTGAGCTGTCAAGCCACTAC; and TLR3 reverse: AAGATATCCTCCAGCCCTCAA. The difference between the expression of TLR3 and the prognosis of TLR3 in KIRC was evaluated with Student's *t*-test and Kaplan–Meier analysis.

### 2.4. Cancer Hallmark Analysis in cBioPortal and GSCALite

TCGA visual tools for genome analysis, including cBioPortal (http://www.cbioportal.org) [23] and GSCALite (http://bioinfo.life.hust.edu.cn/web/GSCALite/) [24], were used to elucidate whether TLRs in KIRC were linked to cancer hallmarks, such as genetic alteration and drug resistance. After obtaining the expression profile of TLRs in KIRC with the threshold as ±2.0 in mRNA expression *z*-scores (RNA Seq V2 RSEM) and protein expression *z*-scores (RPPA), we explored the genetic alteration, coexpression relation, and top 50 most frequently altered neighbour genes of TLRs in cBioPortal. In GSCALite, Spearman correlation analysis was carried out to explore the association between TLRs and well-known cancer-related pathways as well as drug sensitivity. Small molecules and drugs were obtained from the Therapeutics Response Portal (CTRP). A *P* value of 0.05 was set as the threshold. All the analyses were performed using the TCGA KIRC data set (*N* = 537).

### 2.5. Immune Infiltrate Analysis in TIMER

In order to assess the role of TLRs in immune infiltrates, we submitted TLRs into TIMER (https://cistrome.shinyapps.io/timer/), a web portal for the analysis of immune infiltrates in human cancers [25]. In this study, Spearman's correlation analysis was used to explore the association of TLRs with immune cell infiltration and immune biomarker expression in KIRC with the TCGA KIRC data set (*N* = 537). The study included immune cells such as B-cells, CD4+ T-cells, CD8+ T-cells, neutrophils, macrophages, and dendritic cells. It is important to note that the immune biomarkers included in the study had already been reported in previous studies [26–28]. A value of *p* < 0.05 indicated that the difference was statistically significant.

### 2.6. Enrichment Analysis in DAVID, LinkedOmics, and GeneMANIA

In order to verify the functions and underlining mechanisms of TLRs in KIRC, gene ontology (GO) and Kyoto Encyclopedia of Genes and Genomes (KEGG) pathway analysis were performed in DAVID (https://david.ncifcrf.gov/). A value of *p* < 0.05 indicated a statistical significance. We also explored the kinase targets, miRNA targets, and transcription factor targets of TLRs in KIRC using Gene Set Enrichment Analysis (GSEA) in LinkedOmics (http://www.linkedomics.org/) [29]. Again, the *p* value was set as 0.05. After obtaining TLR-associated kinase targets, miRNA targets, and transcription factor targets, we constructed PPI networks based on these targets using GeneMANIA (http://genemania.org/) [30]. All the analyses were performed using the TCGA KIRC data set (*N* = 537).

## 3. Results

### 3.1. The Expression of TLR2, TLR3, TLR7, and TLR8 Was Upregulated in KIRC

**Figure 1:**
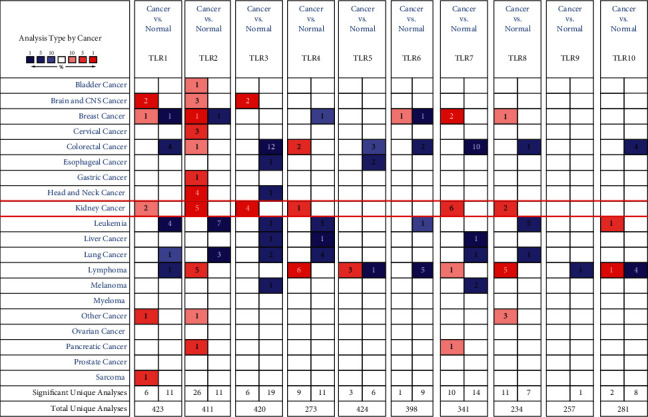
The mRNA level of TLRs in KIRC (Oncomine). The graph shows the number of data sets with statistically significant mRNA overexpression (red) or downregulated expression (blue) of the target genes with a *P* value of 1E−4 and a fold change of 2. The results were generated with “Limma” package of *R* software.

**Figure 2:**
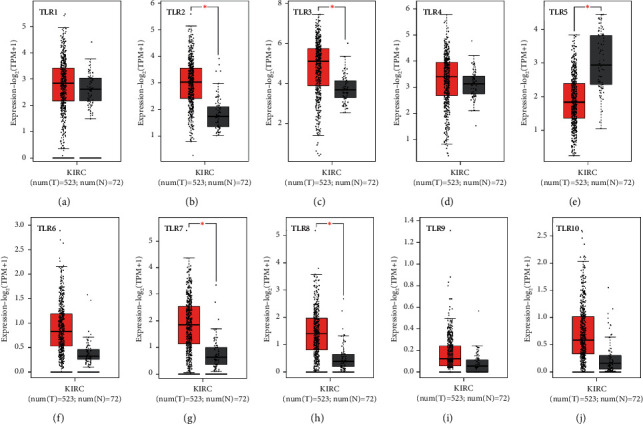
The mRNA level of TLRs in KIRC (GEPIA). Box plots derived from gene expression data from GEPIA comparing the expression of each member of TLRs in KIRC and normal tissues with a *P* value of 0.05. ^*∗*^The results are statistically significant. The difference of gene expression between KIRC tissues and normal tissues was evaluated with Student's *t*-test using the TCGA KIRC data set (*N* = 537).

**Figure 3:**
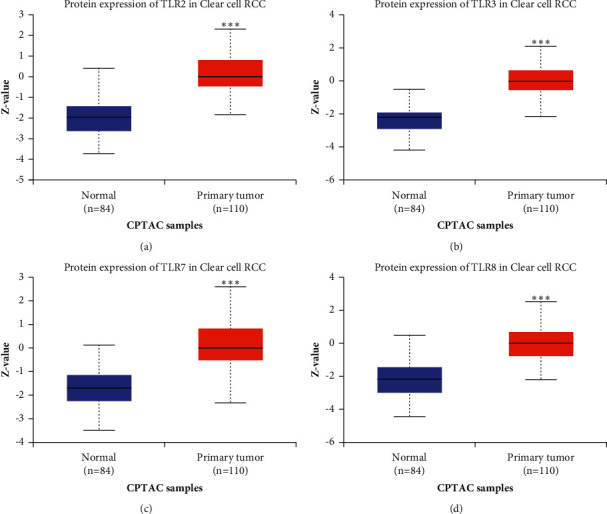
The protein level of TLR2/3/7/8 in KIRC (UALCAN). Box plots derived from protein expression data from UALCAN comparing the expression of TLR2/3/7/8 in KIRC and normal tissues. Data are mean ± SE. ^*∗*^*p* < 0.05; ^*∗∗*^*p* < 0.01; ^*∗∗∗*^*p* < 0.001. The difference of protein expression between KIRC tissues and normal tissues was evaluated with Student's *t*-test using the TCGA KIRC data set (*N* = 537).

We initially detected the expression of TLRs in KIRC at the mRNA and protein levels using Oncomine and UALCAN to explore the potential functions of TLRs in KIRC. According to Oncomine, the mRNA expression of TLRs in pan-cancer analysis revealed the upregulation and downregulation of TLRs in various types of cancers, including KIRC (see Figure 1). In KIRC, the results suggested that the mRNA levels of TLR1, TLR2, TLR3, TLR4, TLR7, and TLR8 were elevated in tumour tissues compared with normal kidney tissues (see Table 1). Specifically, Beroukhim et al. [31] found that TLR1 was upregulated in hereditary and nonhereditary KIRC with an FC of 2.813 and 2.336, respectively (all *p* < 0.05). Interestingly, four data sets demonstrated the upregulation of TLR2 and TLR3 in KIRC tissues in comparison with normal kidney tissues (all FC > 2 and *p* < 0.05) [31–33]. Moreover, TLR5 expression in KIRC was significantly higher than that in normal tissues, and the FC was 2.633 and *p* value was 9.92E − 6 [33]. The results of Gumz et al. [32], Yusenko et al. [33], Beroukhim et al. [31], and Jones et al. [34] indicated that TLR7 expression in KIRC was significantly elevated, and all the FCs were more than 2 (*p* < 0.05). As for the TLR8 expression in KIRC, results of gene expression analysis found an FC of 13.245 of TLR8 in tumour tissues (*p*=1.43E − 7) [33]. In order to further verify this result, we analysed the TCGA KIRC data set in GEPIA. We found that the mRNA expression of TLR2 (see Figure 2(b)), TLR3 (see Figure 2(c)), TLR7 (see Figure 2(g)), and TLR8 (see Figure 2(h)) was increased while TLR5 (see Figure 2(e)) was decreased in KIRC tissues (*p* < 0.05). In conclusion, the mRNA expression of TLR2, TLR3, TLR7, and TLR8 was upregulated in KIRC tissues. We also evaluated the expression of TLR2, TLR3, TLR7, and TLR8 at the protein level using the data from CPTAC. As expected, the results demonstrated the upregulation of TLR2 (see Figure 3(a)), TLR3 (see Figure 3(b)), TLR7 (see Figure 3(c)), and TLR8 (see Figure 3(d)) at the protein level in KIRC, which further confirmed the aforementioned findings. These results indicated that TLRs might play an important role in KIRC.

### 3.2. Genetic Alteration, Well-Known Cancer Hallmark Pathways, and Neighbour Gene Biological Interaction Network of TLRs in KIRC

**Figure 4:**
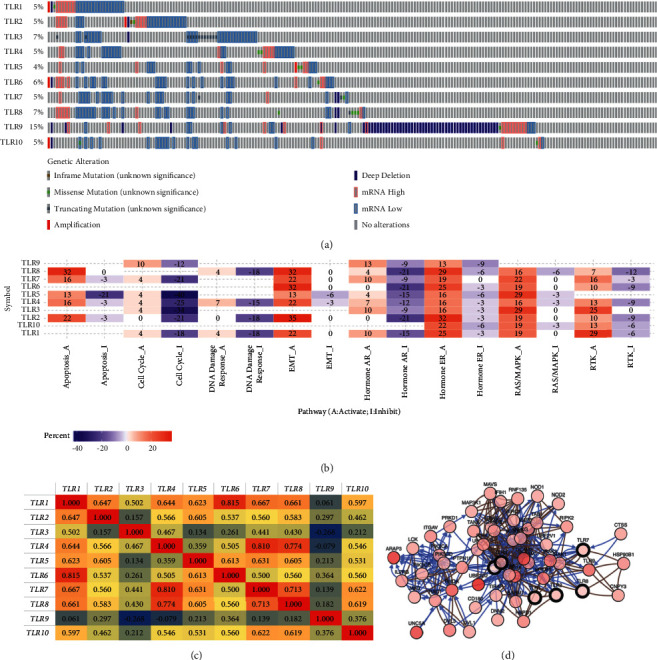
Cancer hallmark analysis of TLRs in KIRC. (a) Summary of genetic alterations of TLRs in KIRC (cBioportal). (b) The activation and inhibition effect of TLRs on well-known cancer-related pathways (GSCALite). The difference of pathway activity score between groups is defined by Student's *t*-test. (c) Correlation heat map of TLRs in KIRC (cBioportal). This analysis was performed with Pearson's correlation test. (d) Gene-gene interaction network of TLRs and 50 most frequently altered neighbouring genes in KIRC. This analysis was performed with Pearson's correlation test using the TCGA KIRC data set (*N* = 537).

Genetic changes are one of the driving factors for the development and progression of KIRC. In order to investigate the role of TLRs in KIRC, we also performed a cancer hallmark analysis. The genetic alterations of TLRs in TCGA KIRC patients mostly comprised inframe mutations, missense mutations, truncating mutations, amplification, deep deletion, mRNA upregulation, and downregulation (see Figure 4(a)). The individual mutated rate of each member of TLRs was 5%, 5%, 7%, 5%, 4%, 6%, 5%, 7%, 15%, and 5% (see Figure 4(a)). It is known that the activation and inhibition of cancer hallmark pathways play a vital role in tumorigenesis and progression. Thus, we analysed the activity of TLRs in well-known cancer hallmark pathways in KIRC, including TSC/mTOR, RTK, RAS/MAPK, PI3K/AKT, hormone ER, hormone AR, EMT, DNA damage response, cell cycle, and apoptosis pathways. After our analysis, we found that TLRs in KIRC were involved in the activation of apoptosis, EMT, hormone ER, RAS/MAPK, and RTK pathways (see Figure 4(b)). On the contrary, TLRs in KIRC were found to be involved in the inhibition of the cell cycle and hormone AR pathways (see Figure 4(b)). Drug resistance is reported as one of the important causes of failure in the treatment of KIRC. Therefore, we also analysed the correlation between the expression of TLRs and drug sensitivity. We found that cells with high expression of TLR3 were resistant to most of the small molecules or drugs, while cells with low expression of TLR7, TLR9, and TLR10 were resistant to most of the small molecules or drugs from CTRP (see Supplementary Figure S1).

A coexpression analysis suggested a moderate to high correlation among the members of TLRs in KIRC, except for TLR9 (see Figure 4(c)). We next wanted to determine the biological interaction network of alterations of TLRs using cBioportal. A total of 50 most frequently altered neighbour genes associated with the alterations of TLRs in KIRC were obtained, including *CNPY3*, *CTSS*, *DNM2*, *DVL1*, *DVL3*, *HSP90B1*, *HSPD1*, *IFIH1*, *IKBKB*, *IKBKE*, *IKBKG*, *IL2RG*, *IRAK1*, *IRAK2*, *ITGAV*, *JAK3*, *LCK*, *LY96*, *MAP3K1*, *MAP3K7*, *MAVS*, *MYD88*, *NOD1*, *NOD2*, *PIK3CA*, *PIK3CB*, *PIK3CD*, *PRKD1*, *PTPN11*, *E2F1*, *RAC1*, *RHOA*, *RIPK1*, *RIPK2*, *RNF135*, *SARM1*, *TAB1*, *TANK*, *TICAM1*, *TICAM2*, *TIPAP*, *UBC*, *UBE2D2*, *UBE2V1*, *UNC5A*, *ARAP3*, *BIRC3*, *CASP8*, *CD14*, and *CD180* (see Figure 4(d)).

### 3.3. TLR-Associated Functional Enrichment Items in KIRC

**Figure 5:**
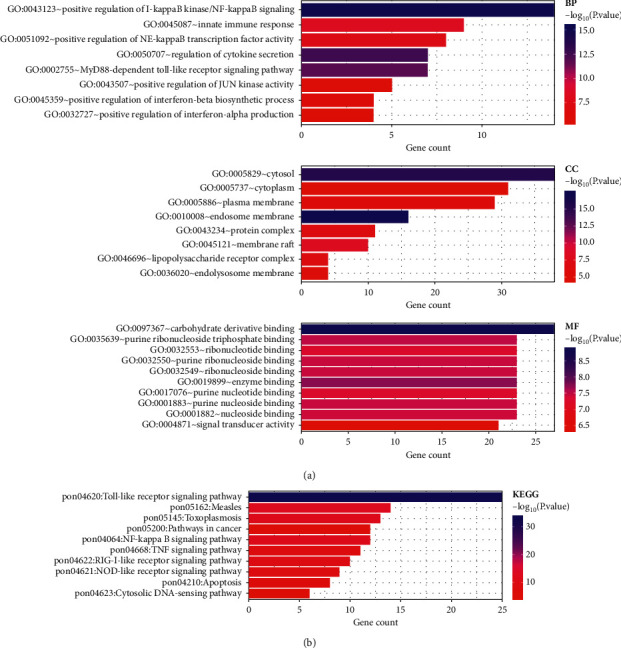
The enrichment analysis of TLRs and 50 most frequently altered neighbouring genes in KIRC (David 6.8). (a) Bar plot of GO enrichment in cellular component terms, biological process terms, and molecular function terms. (b) Bar plot of KEGG-enriched terms. GO and KEGG were conducted with “ggplot2” package in *R* software. *P* value was set as 0.05.

In order to examine the TLR-associated functions in KIRC, we performed an enrichment analysis using DAVID based on TLRs and the top 50 most frequently altered neighbour genes. The GO analysis revealed that TLRs were mainly involved in the positive regulation of I-kappaB kinase/NF-kappaB signalling, regulation of cytokine secretion, innate immune response, carbohydrate derivative binding, enzyme binding, ribonucleoside binding, signal transducer activity, and MyD88-dependent toll-like receptor signalling pathway (see Figure 5(a)). Moreover, KEGG pathway analysis demonstrated that TLRs were mainly involved in toll-like receptor signalling pathway, measles, NF-kappa B signalling pathway, TNF signalling pathway, NOD-like receptor signalling pathway, apoptosis pathway, and cytosolic DNA-sensing pathway (see Figure 5(b)).

### 3.4. TLR3 Served as a Prognostic Biomarker in KIRC

**Figure 6:**
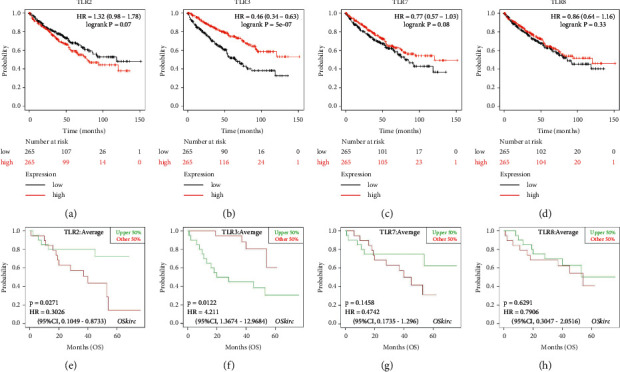
The prognostic value of TLR2/3/7/8 in KIRC. The overall survival curve of TLR2 (a), TLR3 (b), TLR7 (c), and TLR8 (d) in KIRC patients with high/low expression group (Kaplan–Meier plotter). The overall survival curve of TLR2 (e), TLR3 (f), TLR7 (g), and TLR8 (h) in KIRC patients with high/low expression group (OSkirc). Prognosis analysis was performed with the Kaplan–Meier method with the TCGA KIRC data set (*N* = 537). *P* values and hazard ratio (HR) with 95% confidence interval (CI) were generated by log-rank tests and univariate Cox proportional hazard regression.

The aforementioned results demonstrated that the expression of TLR2, TLR3, TLR7, and TLR8 was upregulated at both mRNA and protein levels in KIRC. It would be logical to speculate that these abnormally expressed TLRs may be involved in the progression of KIRC, thus affecting the prognosis of patients. Therefore, these four differentially expressed genes were selected for prognostic analysis using TCGA KIRC data set in Kaplan–Meier and GSE29609 data set in OSkirc, respectively. The analysis findings suggested that KIRC patients with high TLR3 expression were significantly associated with more favourable overall survival (OS) rates (see Figure 6(b), log-rank *p*=5E − 7). However, we found that the expression of TLR2 (see Figure 6(a), log-rank *p*=0.07), TLR7 (see Figure 6(c), log-rank *p*=0.08), and TLR8 (see Figure 6(d), log-rank *p*=0.33) did not affect the prognosis of KIRC patients. We further verified our results using the GSE29609 data set in OSkirc. Interestingly, we found that high expression of TLR2 (see Figure 6(e), log-rank *p*=0.0271) and TLR3 (see Figure 6(f), log-rank *p*=0.0122) was significantly associated with more favourable OS rates. On the contrary, the expression of TLR7 (see Figure 6(g), log-rank *p*=0.1458) and TLR8 (see Figure 6(h), log-rank *p*=0.6291) did not affect the prognosis of KIRC patients, which was consistent with the previous results. These findings suggest that TLR3 may act as a prognostic biomarker in KIRC.

In order to further examine how TLR3 expression affects the prognosis of KIRC patients, we analysed the TLR3 expression and clinical characteristics of KIRC patients in the Kaplan–Meier plotter. After analysing the TCGA KIRC data set, we found that the upregulation of TLR3 in KIRC was linked to a favourable prognosis in male/female patients and high/low mutation burden patients (see Table 2, all *p* < 0.05). Interestingly, the results also suggested that the upregulation of TLR3 in KIRC was linked to a better prognosis of patients in cancer Stages 2 to 4 and tutor Grades 3 to 4 (see Table 2, All *p* < 0.05). However, there was no difference in the prognosis between KIRC patients in cancer Stage 1 and tumour Grades 1 to 2 with high and low TLR3 expressions (all *p* > 0.05). These results indicated that TLR3 expression might affect the prognosis of KIRC patients with advanced cancer stage and high tumour grade.

### 3.5. TLR3 May Help Detect Patients with KIRC

**Figure 7:**
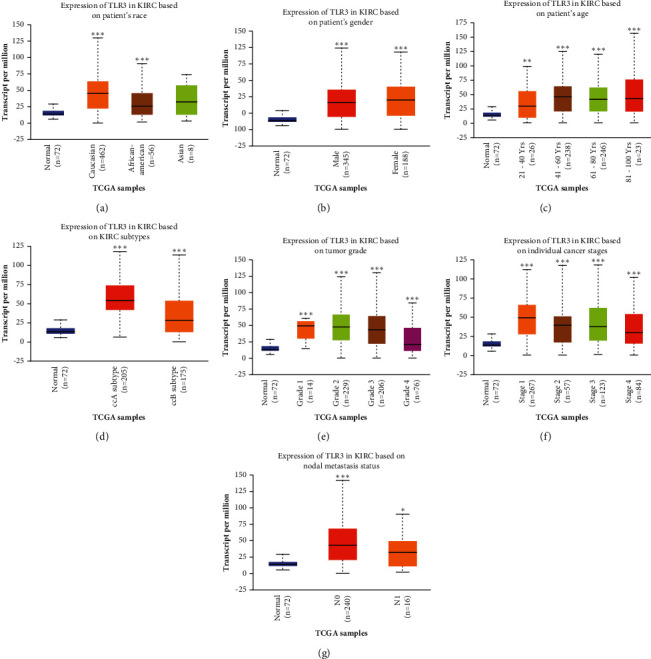
The mRNA expression of TLR3 in subgroups of patients with KIRC (UALCAN). (a) TLR3 expression in normal and KIRC (Caucasian, African-American, or Asian) samples. (b) TLR3 expression in normal and KIRC (male or female) samples. (c) TLR3 expression in normal and KIRC (21–40, 41–60, 61–80, or 81–100 years old) samples. (d) TLR3 expression in normal and KIRC (ccA subtype or ccB subtype) samples. (e) TLR3 expression in normal and KIRC (Grades 1, 2, 3, or 4) samples. (f) TLR3 expression in normal and KIRC (Stages 1, 2, 3, or 4) samples. (g) TLR3 expression in normal and KIRC (with or without nodal metastasis) samples. Data are mean ± SE. ^*∗*^*p* < 0.05; ^*∗∗*^*p* < 0.01; ^*∗∗∗*^*p* < 0.001. The difference of TLR3 mRNA expression among groups was analyzed with the rank-sum test using the TCGA KIRC data set (*N* = 537).

**Figure 8:**
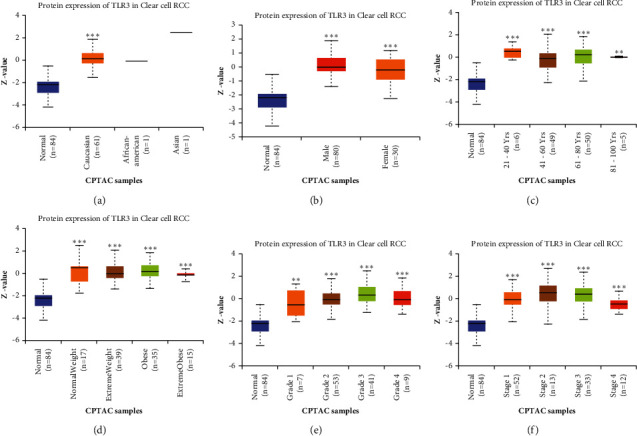
The protein expression of TLR3 in subgroups of patients with KIRC (UALCAN). (a) TLR3 expression in normal and KIRC (Caucasian, African-American, or Asian) samples. (b) TLR3 expression in normal and KIRC (male or female) samples. (c) TLR3 expression in normal and KIRC (21–40, 41–60, 61–80, or 81–100 years old) samples. (d) TLR3 expression in normal and KIRC (normal-weight, extreme-weight, obese, or extreme-obese) samples. (e) TLR3 expression in normal and KIRC (Grades 1, 2, 3, or 4) samples. (f) TLR3 expression in normal and KIRC (Stages 1, 2, 3, or 4) samples. Data are mean ± SE. ^*∗*^*p* < 0.05; ^*∗∗*^*p* < 0.01; ^*∗∗∗*^*p* < 0.001. The difference of TLR3 protein expression among groups was analysed with the rank-sum test using the TCGA KIRC data set (*N* = 537).

The aforementioned results revealed that TLR3 was upregulated at mRNA and proteins levels in KIRC and served as a prognostic biomarker. Therefore, TLR3 was selected for further analysis, and its diagnosis value in KIRC was explored. The mRNA and protein expression of TLR3 in different subgroups of patients with KIRC was analysed, which revealed an increased TLR3 mRNA expression in patients with KIRC compared with the healthy controls based on race, gender, age, subtypes, tumour grade, cancer stages, and nodal metastasis status (see Figure 7, all *p* < 0.05). Similar results were obtained in a protein-level analysis. The results indicated that TLR3 protein expression was increased in patients with KIRC compared with the healthy controls in subgroup analyses based on race, gender, age, weight, tumour grade, and cancer stages (see Figure 8, all *p* < 0.05). These findings suggested that TLR3 might be involved in tumour progression and help detect patients with KIRC.

### 3.6. TLR3 Was Upregulated and Associated with Poor Overall Survival in KIRC

**Figure 9:**
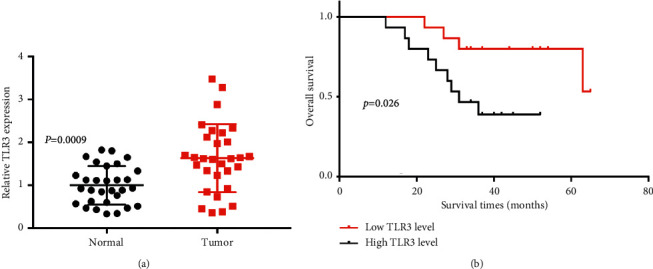
Validation of the expression and prognostic value of TLR3 in KIRC. (a) The relative expression of TLR3 in KIRC tissues and normal tissues. (b) The overall survival in KIRC patients with high and low expression of TLR3. The difference between the expression of TLR3 and the prognosis of TLR3 in KIRC was evaluated with Student's *t*-test and Kaplan–Meier analysis, respectively. These analyses were performed with clinical KIRC dataset (*N* = 30).

We detected the expression and prognostic value of TLR3 in KIRC using a clinical tissue specimen. As expected, the results revealed that TLR3 expression was increased in KIRC tissues compared with normal tissues (see Figure 9(a), *p*=0.0009). Moreover, prognosis analysis indicated that breast cancer patients with high TLR3 levels had a poor overall survival (see Figure 9(b), *p*=0.026). These findings were consistent with the aforementioned data.

### 3.7. TLR3 Was Associated with Tumour Immune Infiltration in KIRC

**Figure 10:**
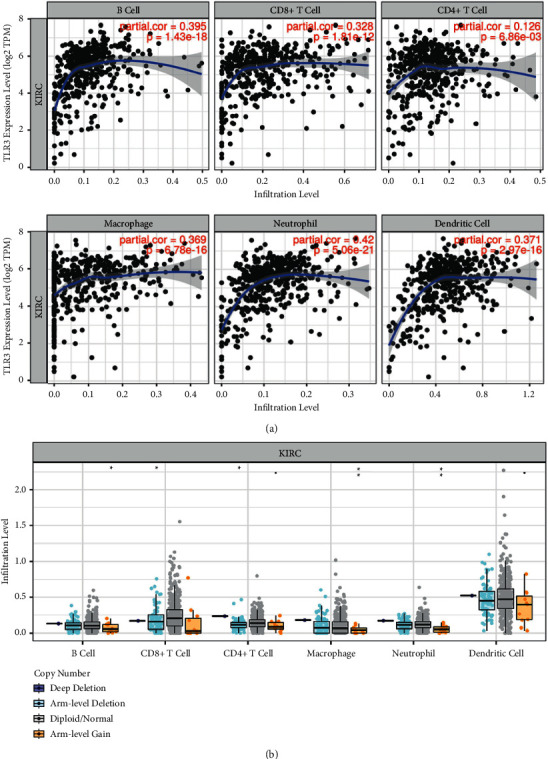
The correlation between TLR3 and immune infiltration (TIMER). (a) The correlation between TLR3 expression and the abundance of CD8+ T-cells, CD4+ T-cells, macrophages, neutrophils, and dendritic cells. This analysis was performed with Pearson's correlation test using KIRC TCGA data set (*N* = 537). (b) The correlation between SCNA of TLRs and immune cell infiltration. SCNA, somatic copy number alterations. The infiltration level for each SCNA category is compared with the normal using a two-sided Wilcoxon rank-sum test using the KIRC TCGA data set (*N* = 537). ^*∗*^*p* < 0.05; ^*∗∗*^*p* < 0.01; ^*∗∗∗*^*p* < 0.001.

Previous studies have emphasised the significance of tumour immune infiltration in the prognosis of renal cancer [7, 35]. It has also been suggested that TLRs may be significantly associated with immune response in tumour progression. As expected, our enrichment analysis revealed that TLRs were involved in the immune response. We further explored the association between mRNA expression of TLRs and immune infiltration in KIRC. The findings indicated that TLR3 expression was positively linked to the infiltration of B-cells (*p*=1.43E − 18), CD8+ T-cells (*p*=1.81E − 12), CD4+ T-cells (*p*=6.86E − 3), macrophages (*p*=6.78E − 16), neutrophils (*p*=5.06E − 21), and dendritic cells (*p*=2.97E − 16) (see Figure 10(a)). Interestingly, copy number alteration of TLR3 could suppress immune infiltration to a certain extent (see Figure 10(b)).

We also investigated thoroughly whether TLR3 expression was correlated with immune biomarkers of different immune cells in KIRC. A significant correlation was obtained between TLR3 expression and most of the immune biomarkers in KIRC after tumour purity modulation. Specifically, TLR3 in KIRC was positively correlated with CD8A and CD8B in CD8+ T-cells; CD3D, CD3E, and CD2 in CD19 T-cells; CD86 and CD115 in monocytes; CCL2, CD68, and IL10 in TAM cells; and NOS2 in M1 macrophages (see Table 3, all *p* < 0.05). All the biomarkers of M2 macrophages (CD163, VSIG4, and MS4A4A) and neutrophils (CD66b, CD11b, and CCR7) showed a positive association with TLR3 in KIRC (see Table 3, all *p* < 0.05).

Moreover, the expression of KIR2DL1, KIR2DL3, KIR3DL1, KIR3DL2, and KIR2DS4 in natural killer cells was positively correlated with TLR3 in KIRC (see Table 3, all *p* < 0.05). A positive correlation was obtained between all the biomarkers of dendritic cells as well as Th1 and TLR3 in KIRC, except for ITGAX (see Table 3, all *p* < 0.05). The results also revealed that TLR3 in KIRC was positively correlated with STAT6 and STAT5A in Th2 cells, BCL6 in Tfh cells, and STAT3 in Th17 cells (see Table 3, all *p* < 0.05). In Treg cells, three biomarkers (CCR8, STAT5B, and TGFB1) were positively correlated with TLR3 in KIRC (see Table 3, all *p* < 0.05). Interestingly, the level of CTLA4 and TIM-3 of T-cell exhaustion was positively correlated with TLR3 in KIRC (see Table 3, all *p* < 0.05). Thus, TLR3 was found to be associated with tumour immune infiltration in KIRC and suggested to play a vital role in immune escape in the KIRC microenvironment.

### 3.8. Prognostic Analysis of TLR3 in KIRC Based on Immune Cells

**Figure 11:**
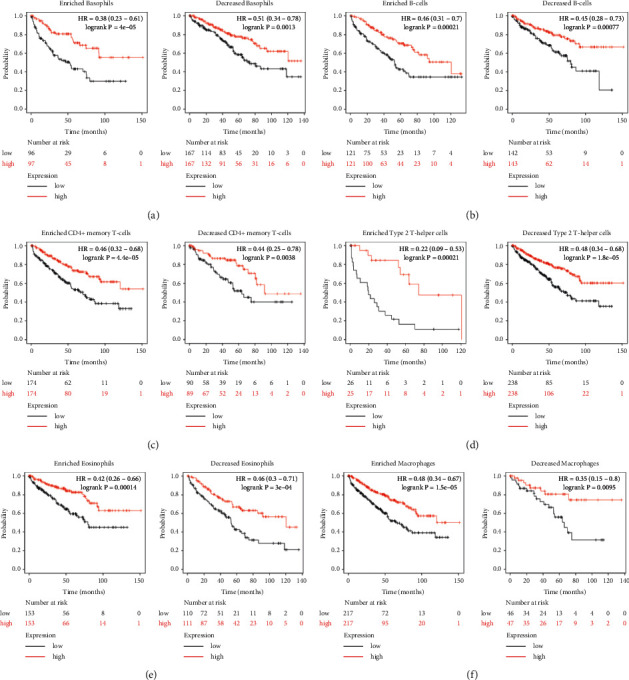
Prognostic value of TLR3 in KIRC based on immune cells subgroups (Kaplan–Meier plotter). Prognosis analysis was performed with the Kaplan–Meier method. *P* values and hazard ratio (HR) with 95% confidence interval (CI) were generated by log-rank tests and univariate Cox proportional hazard regression using the KIRC TCGA data set (*N* = 537).

**Figure 12:**
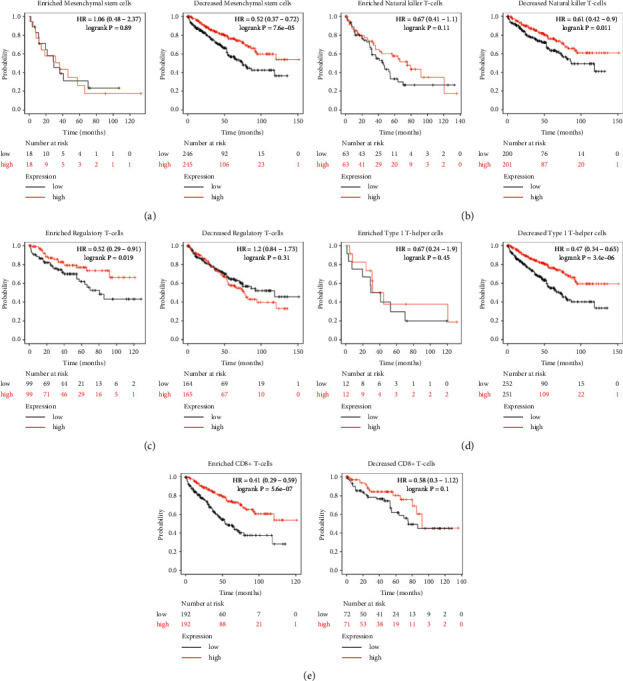
Prognostic value of TLR3 in KIRC based on immune cell subgroups (Kaplan–Meier plotter). Prognosis analysis was performed with the Kaplan–Meier method. *P* values and hazard ratio (HR) with 95% confidence interval (CI) were generated by log-rank tests and univariate Cox proportional hazards regression using the KIRC TCGA data set (*N* = 537).

The aforementioned results suggested that TLR3 in KIRC was associated with the favourable prognosis and immune infiltration. Moreover, immune cell infiltration was significantly associated with the outcome of patients with renal cancers [5, 36]. Therefore, we further performed a prognostic analysis of TLR3 in KIRC based on immune cells to verify whether TLR3 expression could affect the prognosis because of immune infiltration. The results indicated that high TLR3 expression was present in enriched/decreased basophil cohort (see Figure 11(a)), enriched/decreased B-cell cohort (see Figure 11(b)), enriched/decreased CD4+ memory T-cell cohort (see Figure 11(c)), enriched/decreased Type-2 T-helper cell cohort (see Figure 11(d)), enriched/decreased eosinophils (see Figure 11(e)), and enriched/decreased macrophages (see Figure 11(f)) (all *p* < 0.05). Interestingly, the results also indicated that high TLR3 expression in decreased mesenchymal stem cell cohort (see Figure 12(a)), decreased natural killer T-cell cohort (see Figure 12(b)), enriched regulatory T-cell cohort (see Figure 12(c)), decreased Type-1 T-helper cell cohort (see Figure 12(d)), and enriched CD8+ T-cells (see Figure 12(e)) was associated with favourable prognosis. However, there was no significant correlation between the high TLR3 and the prognosis of KIRC in enriched mesenchymal stem cell cohort (see Figure 12(a)), enriched natural killer T-cell cohort (see Figure 12(b)), decreased regulatory T-cell cohort (see Figure 12(c)), enriched Type-1 T-helper cell cohort (see Figure 12(d)), and decreased CD8+ T-cells (see Figure 12(e)). These findings suggest that TLR3 may affect the prognosis of KIRC patients in part due to immune infiltration.

### 3.9. TLR3-Associated Kinase, miRNA, or Transcription Factor Targets in KIRC

**Figure 13:**
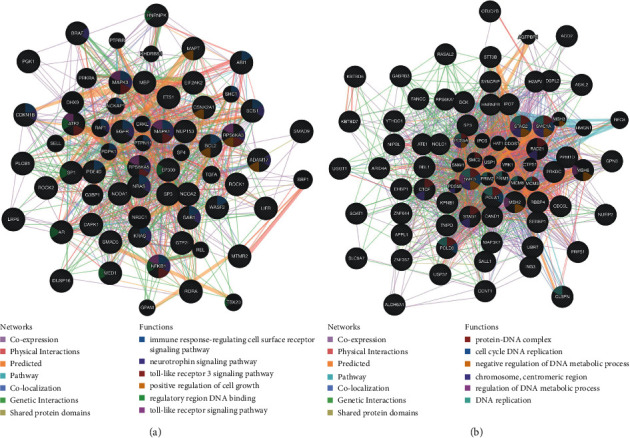
PPI network of TLR3-associated target networks (GeneMANIA). (a) PPI network of TLR3-associated kinase target MAPK1 networks. (b) PPI network of TLR3-associated transcription factor E2F1 networks. Different colors of the network edge indicate the bioinformatics methods applied: coexpression, website prediction, pathway, physical interactions, and colocalization. The different colors for the network nodes indicate the biological functions of the set of enrichment genes.

To further examine the underlining mechanisms of TLR3 in the tumorigenesis and progression of KIRC, we analysed TLR3-associated kinase, miRNA, and transcription factor targets in KIRC with GSEA in LinkedOmics. We found that the top 5 most significant TLR3-associated kinase targets in KIRC were MAPK1, MAPK3, CAMKK2, RIPK2, and HCK (see Table 4, all *p* < 0.05). The PPI network based on the correlated genes of kinases MAPK1 constructed by GeneMANIA indicated that kinases MAPK1 were mainly involved in immune response, neurotrophin signalling pathway, toll-like receptor signalling pathway, and positive regulation of cell growth (see Figure 13(a)). As for transcription factor target analysis, the results identified V$E2F1_Q6, V$E2F_Q2, V$LFA1_Q6, V$SOX5_01, and V$FOXO4_02 as the TLR3-associated targets in KIRC (see Table 4, all *p* < 0.05). The PPI network based on the correlated genes of transcription factor E2F1 constructed by GeneMANIA revealed that the transcription factor E2F1 was mainly involved in protein-DNA complex, cell cycle, DNA replication, and regulation of DNA metabolic processes and chromosomes (see Figure 13(b)). Moreover, the 5 TLR3-associated miRNA targets were identified as (TAATGTG) MIR-323, (TGCTTTG) MIR-330, (ATATGCA) MIR-448, (ATAGGAA) MIR-202, and (ATTCTTT) MIR-186 (see Table 4, all *p* < 0.05). The PPI network based on the correlated genes of transcription factor E2F1 constructed by GeneMANIA found that MIR-323 was mainly involved in the positive regulation of cell cycle processes and ubiquitin-protein ligase activity (see Supplementary Figure S2).

## 4. Discussion

Accumulating evidence suggests that TLRs are involved in the tumorigenesis and progression of cancers and act as prognostic biomarkers in cancers and many other diseases [37–39]. TLRs are reported to regulate metabolic reprogramming in the tumour microenvironment, making them promising targets for cancer immunotherapy [40]. However, the precise functions of TLRs in KIRC and their association with immune infiltration have not been investigated previously. Thus, we conducted our study to shed light on this understudied area.

We first performed expression analysis, which indicated that the expressions of TLR2, TLR3, TLR7, and TLR8 were increased at mRNA and protein levels. Further prognosis analysis revealed that high TLR3 expression in KIRC was associated with a favourable prognosis in TCGA and GEO cohorts. Moreover, ACE2 was identified as a potential prognostic biomarker for KIRC, predicting favourable outcome. Importantly, TLR3 has also been suggested as a potential biomarker in other types of cancers and diseases. For example, Yuan et al. revealed that TLR3 acted as a prognostic biomarker in hepatocellular carcinoma and suppressed tumour cell proliferation [41]. TLR3 was also reported as a potential biomarker for advanced oral cancer and associated with worse overall survival [42]. Moreover, TLR3 was found as a marker predicting the risk of doxorubicin-induced heart failure [39].

Our results revealed that TLR3 was upregulated in KIRC and served as a prognostic biomarker. Moreover, high expression of TLR3 in cells was found to cause resistance to most of the small molecules or drugs. Therefore, TLR3 was selected, specifically, among all the members of TLRs for further examination. Previous studies indicated that immune‐cell infiltration could regulate tumour progression and metastasis, thus affecting the prognosis of patients [3, 43, 44]. TLRs were also associated with tumour microenvironment and cancer immunotherapy [40]. Thus, we decided to evaluate the correlation between TLR3 and immune infiltration in KIRC as well as the prognosis of patients. As expected, TLR3 in KIRC showed a strong association with a myriad of immune cells, including B-cells, CD4+ T-cells, CD8+ T-cells, macrophages, neutrophils, and dendritic cells. A strong association was also found between TLR3 and the expression of most of the immune biomarker sets. Thus, we concluded that TLR3 might affect the prognosis of KIRC patients in part due to immune infiltration. Importantly, these immune cells and biomarkers were involved in tumour progression and as biomarkers for prognosis and therapy of renal cancers. Xiong et al. found that the tumour microenvironment in B-cells was associated with dismal survival and worse treatment response in KIRC [45]. Another study found that KIRC patients with the absence of fully functional mature dendritic cells had an increased risk of disease progression [46]. Moreover, CTLA4, an immunomodulatory molecule, was found to act as a prognostic biomarker, predicting poor overall survival in KIRC [7]. Thus, TLR3 may be involved in immune escape in the KIRC microenvironment, thus affecting the prognosis of patients.

To further investigate the underlining mechanisms of TLR3 on the tumorigenesis and progression of KIRC, we analysed TLR3-associated kinases in KIRC. The results identified MAPK1, MAPK3, CAMKK2, RIPK2, and HCK as potential targets. It has been suggested that genomic instability and mutagenesis could result in tumour genesis and progression while kinase could stabilise and repair genomic DNA [47]. Furthermore, MAPK1 was reported to be involved in tumour genesis and progression in many cancers, including endometrial cancer and cervical cancer [48, 49]. Our study found that TLR3-associated kinases and MAPK1 were mainly involved in immune response, modulation of the toll-like receptor signalling pathway, and positive regulation of cell growth. These are consistent with the previous studies that found MAPK1 to regulate the cell cycle and be associated with immune infiltration in KIRC [5, 50]. Therefore, we hypothesised that TLR3 might regulate immune infiltration and tumour progression in KIRC via kinase MAPK1. However, further studies should be conducted to confirm this hypothesis.

Constant proliferation and abnormal invasion caused by transcriptional dysregulation and cell cycle disorder have been identified as the basic characteristics of the KIRC. Transcription factors are a category of regulators playing a significant part in mRNA transcription and cell cycle control [51]. Our results highlighted the E2F family, particularly E2F1, as the TLR3-associated transcription factor target in KIRC.

Interestingly, a previous study revealed that E2F1 could regulate the innate immune receptor TLR3 in epithelial cells [52]. Furthermore, E2F1 was found to play a vital role in the regulation of tumour cell proliferation, invasion, and metastasis in KIRC [53, 54]. In our study, we found that TLR3-associated transcription factor target E2F1 was mainly involved in protein-DNA complex, cell cycle DNA replication, and regulation of DNA metabolic process and chromosomes. These findings are consistent with the fact that E2F1 could regulate the cell cycle in progression of KIRC [54]. Therefore, it would be logical to speculate that TLR3 may regulate the cell cycle and progression of KIRC via E2F1. However, future studies to test this hypothesis are warranted.

## 5. Conclusion

In conclusion, our results indicate that TLR3 is a prognostic biomarker and associated with immune infiltration in KIRC. This lays a foundation for future research on the role of TLR3 in the carcinogenesis and progression of KIRC.

## Figures and Tables

**Table 1:** The mRNA levels of TLRs in KIRC (Oncomine).

TLR	Type	Fold change	*P* value	*t*-test	Reference
TLR1	Hereditary clear cell renal cell carcinoma	2.813	1.91E−7	7.773	PMID: 19470766
	Nonhereditary clear cell renal cell carcinoma	2.336	6.45E−6	5.235	PMID: 19470766

TLR2	Clear cell renal cell carcinoma	11.321	1.25E−9	11.166	PMID: 17699851
	Clear cell renal cell carcinoma	8.793	6.75E−7	9.237	PMID: 19445733
	Hereditary clear cell renal cell carcinoma	4.242	2.09E−8	8.561	PMID: 19470766
	Nonhereditary clear cell renal cell carcinoma	4.789	2.12E−8	10.118	PMID: 19470766

TLR3	Clear cell renal cell carcinoma	7.203	2.53E−8	7.023	PMID: 17699851
	Clear cell renal cell carcinoma	9.081	1.26E−5	9.801	PMID: 19445733
	Hereditary clear cell renal cell carcinoma	7.493	1.16E−14	7.493	PMID: 19470766
	Nonhereditary clear cell renal cell carcinoma	4.939	1.66E−11	4.939	PMID: 19470766

TLR4	Clear cell renal cell carcinoma	2.633	9.92E−6	7.051	PMID: 19445733

TLR5	NA	NA	NA	NA	NA

TLR6	NA	NA	NA	NA	NA

TLR7	Clear cell renal cell carcinoma	13.936	3.84E−5	6.110	PMID: 17699851
	Clear cell renal cell carcinoma	11.984	6.54E−5	8.635	PMID: 19445733
	Hereditary clear cell renal cell carcinoma	2.493	2.82E−7	6.165	PMID: 19470766
	Nonhereditary clear cell renal cell carcinoma	2.243	3.16E−7	6.787	PMID: 19470766
	Clear cell renal cell carcinoma	3.348	1.09E−10	8.345	PMID: 16115910

TLR8	Clear cell renal cell carcinoma	13.245	1.43E−7	13.245	PMID: 19445733

TLR9	NA	NA	NA	NA	NA

TLR10	NA	NA	NA	NA	NA

**Table 2:** Correlation of TLR3 expression and the prognosis of KIRC with different clinicopathological factors (Kaplan–Meier plotter).

Pathological parameters	Overall survival		
	*N*	Hazard ratio	*P* value
*Stage status*			
1	398	0.6 (0.33–1.09)	0.088
2	183	0.11 (0.01–0.84)	0.01
3	332	0.47 (0.26–0.84)	0.0093
4	188	0.38 (0.22–0.67)	5*e*^−4^

*Gender*			
Female	284	0.36 (0.21–0.62)	9.5*e*^−5^
Male	948	0.39 (0.26–0.56)	3*e*^−7^

*Race*			
White	690	0.41 (0.3–0.57)	1.1*e*^−8^
Asian	8	NA	NA
Black/African-American	111	0.27 (0.06–1.25)	0.073

*Tumor grade*			
1	14	NA	NA
2	340	0.6 (0.31–1.16)	0.12
3	585	0.33 (0.2–0.53)	2.1*e*^−6^
4	174	0.4 (0.22–0.71)	0.0013

*Mutation burden*			
High	246	0.41 (0.23–0.72)	0.0015
Low	437	0.34 (0.15–0.74)	0.0042

**Table 3:** Correlation analysis between TLR3 and gene biomarkers of immune cells in KIRC (TIMER).

Description	Biomarkers	None		Purity	
		Cor	*P* value	Cor	*P* value
CD8+ T-cells	CD8A	0.232	^*∗∗∗*^	0.213	^*∗∗∗*^
	CD8B	0.191	^*∗∗∗*^	0.171	^*∗∗∗*^

T-cells (general)	CD3D	0.134	^*∗∗*^	0.116	^*∗*^
	CD3E	0.161	^*∗∗∗*^	0.141	^*∗∗*^
	CD2	0.221	^*∗∗∗*^	0.209	^*∗∗∗*^

B-cells	CD19	−0.087	^*∗*^	−0.117	^*∗*^
	CD79A	−0.045	0.305	−0.09	0.0526

Monocyte	CD86	0.351	^*∗∗∗*^	0.349	^*∗∗∗*^
	CD115 (CSF1R)	0.328	^*∗∗∗*^	0.302	^*∗∗∗*^

TAM	CCL2	0.085	^*∗*^	0.093	^*∗*^
	CD68	0.359	^*∗∗∗*^	0.317	^*∗∗∗*^
	IL10	0.292	^*∗∗∗*^	0.267	^*∗∗∗*^

M1 macrophages	INOS (NOS2)	0.308	^*∗∗∗*^	0.263	^*∗∗∗*^
	IRF5	0.106	^*∗*^	0.085	0.0678
	COX2 (PTGS2)	0.069	0.111	0.086	0.0649

M2 macrophages	CD163	0.423	^*∗∗∗*^	0.392	^*∗∗∗*^
	VSIG4	0.257	^*∗∗∗*^	0.203	^*∗∗∗*^
	MS4A4A	0.363	^*∗∗∗*^	0.343	^*∗∗∗*^

Neutrophils	CD66b (CEACAM8)	0.114	^*∗∗*^	0.104	^*∗*^
	CD11b (ITGAM)	0.347	^*∗∗∗*^	0.328	^*∗∗∗*^
	CCR7	0.161	^*∗∗∗*^	0.125	^*∗∗*^

Natural killer cells	KIR2DL1	0.174	^*∗∗∗*^	0.144	^*∗*^
	KIR2DL3	0.152	^*∗∗∗*^	0.125	^*∗∗*^
	KIR2DL4	0.091	^*∗*^	0.084	0.0714
	KIR3DL1	0.215	^*∗∗∗*^	0.189	^*∗∗∗*^
	KIR3DL2	0.138	^*∗∗*^	0.122	^*∗∗*^
	KIR3DL3	0.033	0.446	0.045	0.339
	KIR2DS4	0.116	^*∗∗*^	0.107	^*∗*^

Dendritic cells	HLA-DPB1	0.372	^*∗∗∗*^	0.354	^*∗∗∗*^
	HLA-DQB1	0.299	^*∗∗∗*^	0.276	^*∗∗∗*^
	HLA-DRA	0.463	^*∗∗∗*^	0.452	^*∗∗∗*^
	HLA-DPA1	0.474	^*∗∗∗*^	0.473	^*∗∗∗*^
	BDCA-1 (CD1C)	0.217	^*∗∗∗*^	0.184	^*∗∗∗*^
	BDCA-4 (NRP1)	0.543	^*∗∗∗*^	0.51	^*∗∗∗*^
	CD11c (ITGAX)	0.048	0.267	0.049	0.292

Th1	T-bet (TBX21)	0.164	^*∗∗∗*^	0.139	^*∗∗*^
	STAT4	0.206	^*∗∗∗*^	0.187	^*∗∗∗*^
	STAT1	0.495	^*∗∗∗*^	0.479	^*∗∗∗*^
	IFN-g (IFNG)	0.173	^*∗∗∗*^	0.156	^*∗∗∗*^
	TNF-a (TNF)	0.133	^*∗∗*^	0.102	^*∗*^

Th2	GATA3	−0.068	0.119	−0.053	0.26
	STAT6	0.45	^*∗∗∗*^	0.435	^*∗∗∗*^
	STAT5A	0.307	^*∗∗∗*^	0.304	^*∗∗∗*^
	IL13	−0.089	^*∗*^	−0.079	0.09

Tfh	BCL6	0.179	^*∗∗∗*^	0.166	^*∗∗∗*^
	IL21	0.001	0.989	−0.018	0.698

Th17	STAT3	0.563	^*∗∗∗*^	0.539	^*∗∗∗*^
	IL17A	−0.055	0.207	−0.037	0.425

Treg	FOXP3	0.054	0.214	0.039	0.404
	CCR8	0.293	^*∗∗∗*^	0.289	^*∗∗∗*^
	STAT5B	0.63	^*∗∗∗*^	0.618	^*∗∗∗*^
	TGFb (TGFB1)	0.167	^*∗∗∗*^	0.146	^*∗∗*^

T-cell exhaustion	PD-1 (PDCD1)	0.07	0.107	0.054	0.243
	CTLA4	0.101	^*∗*^	0.096	^*∗*^
	LAG3	0.083	0.0555	0.07	0.135
	TIM-3 (HAVCR2)	0.413	^*∗∗∗*^	0.393	^*∗∗∗*^
	GZMB	−0.016	0.706	−0.055	0.237

**Table 4:** The kinase and transcription factor-target networks of TLR3 in KIRC (LinkedOmics).

Enriched category	Geneset	LeadingEdgeNum	FDR
Kinase target	Kinase_MAPK1	44	0
	Kinase_MAPK3	42	0
	Kinase_CAMKK2	43	0.008
	Kinase_RIPK2	20	0.009
	Kinase_HCK	25	0.009

miRNA target	TAATGTG, MIR-323	53	0
	TGCTTTG, MIR-330	110	0
	ATATGCA, MIR-448	78	0
	ATAGGAA, MIR-202	46	0
	ATTCTTT, MIR-186	95	0.007

Transcription factor target	V$E2F1_Q6	58	0
	V$E2F_Q2	46	0
	V$LFA1_Q6	73	0
	V$SOX5_01	72	0
	V$FOXO4_02	56	0

## Data Availability

The analysed data sets generated during the study are available from the corresponding author on reasonable request.

## Abbreviations

KIRC: Clear cell renal cell carcinoma
TLR3: Toll-like receptors 3
GO: Gene Ontology
KEGG: Kyoto Encyclopedia of Genes and Genomes
BP: Biological processes
CC: Cellular components
MF: Molecular functions
GSEA: Gene Set Enrichment Analysis
OS: Overall survival.

## Supplementary Materials

Supplementary Materials *Supplementary Figure 1.* Drug sensitivity analysis of TLRs in KIRC. Spearman's correlation represents the gene expression correlates with the drug. The positive correlation means that the gene high expression is resistant to the drug, vice versa. *Supplementary Figure 2*. PPI network of TLR3-associated target MIR-323 networks (GeneMANIA). Different colors of the network edge indicate the bioinformatics methods applied: coexpression, website prediction, pathway, physical interactions, and colocalization. The different colors for the network nodes indicate the biological functions of the set of enrichment genes.
