# Supplementary figures and images for: TLR3 Serves as a Prognostic Biomarker and Associates with Immune Infiltration in the Renal Clear Cell Carcinoma Microenvironment
Source: J Oncol. 2021 Sep 6;2021:3336770. doi: 10.1155/2021/3336770 (PMC8440088; doi:10.1155/2021/3336770)

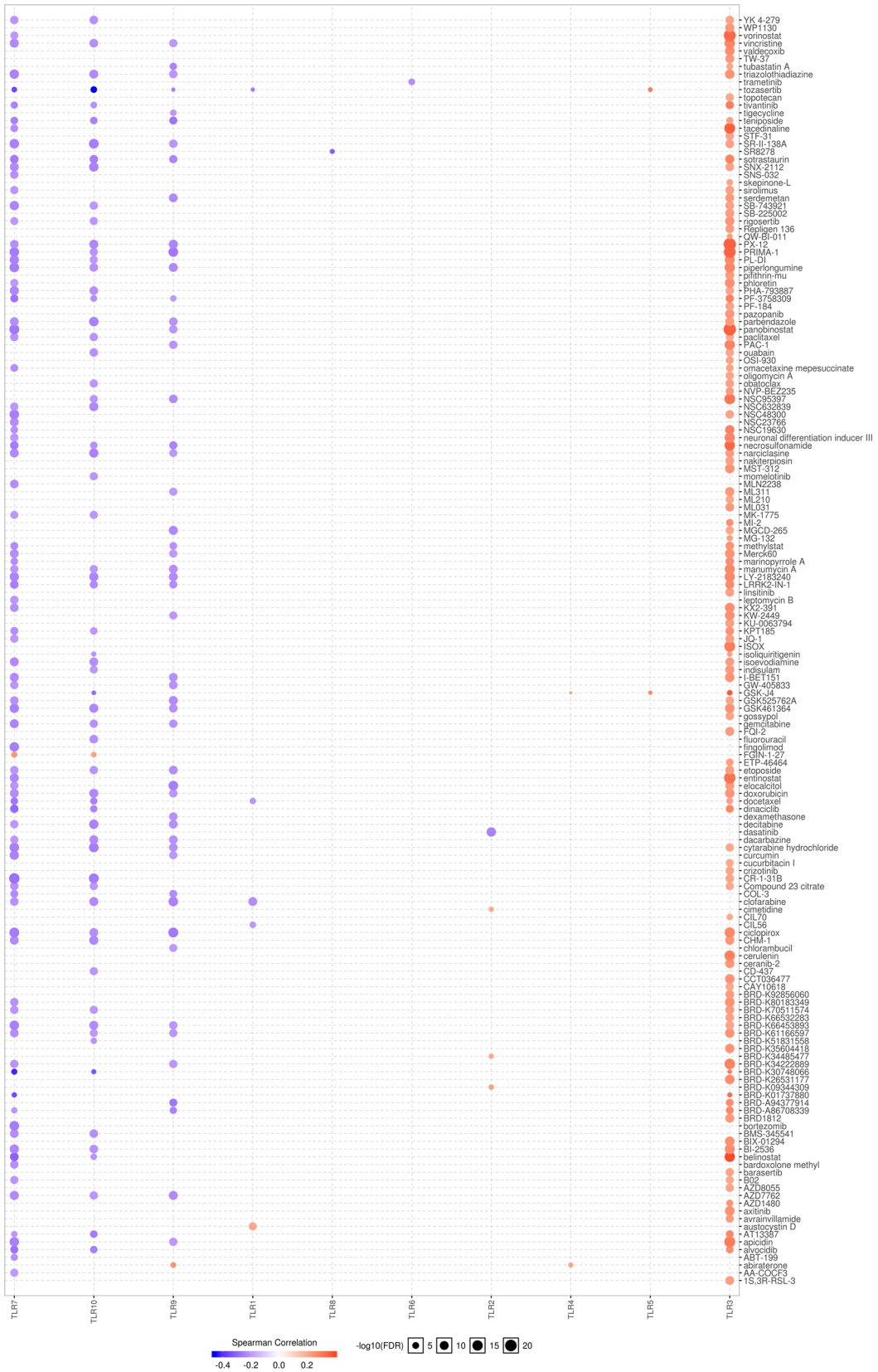

Supplement: Supplementary Materials — Supplementary Figure 1. Drug sensitivity analysis of TLRs in KIRC. Spearman's correlation represents the gene expression correlates with the drug. The positive correlation means that the gene high expression is resistant to the drug, vice versa. Supplementary Figure 2. PPI network of TLR3-associated target MIR-323 networks (GeneMANIA). Different colors of the network edge indicate the bioinformatics methods applied: coexpression, website prediction, pathway, physical interactions, and colocalization. The different colors for the network nodes indicate the biological functions of the set of enrichment genes. [file 3336770.f1.zip › 3336770.f1/Supplementary Fig 1.pdf]

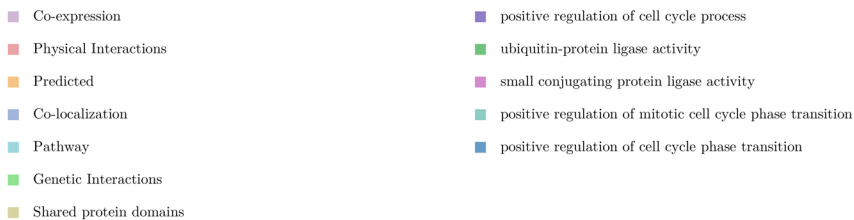

Supplement: Supplementary Materials — Supplementary Figure 1. Drug sensitivity analysis of TLRs in KIRC. Spearman's correlation represents the gene expression correlates with the drug. The positive correlation means that the gene high expression is resistant to the drug, vice versa. Supplementary Figure 2. PPI network of TLR3-associated target MIR-323 networks (GeneMANIA). Different colors of the network edge indicate the bioinformatics methods applied: coexpression, website prediction, pathway, physical interactions, and colocalization. The different colors for the network nodes indicate the biological functions of the set of enrichment genes. [file 3336770.f1.zip › 3336770.f1/Supplementary Fig 2.pdf]
